# Additive Anticonvulsant Profile and Molecular Docking Analysis of 5,5′-Diphenylhydantoin Schiff Bases and Phenytoin

**DOI:** 10.3390/biomedicines11112912

**Published:** 2023-10-27

**Authors:** Jana Tchekalarova, Petar Todorov, Miroslav Rangelov, Tsveta Stoyanova, Nadezhda Todorova

**Affiliations:** 1 Institute of Neurobiology, Bulgarian Academy of Sciences, 1113 Sofia, Bulgaria; tzafti@abv.bg; 2 Department of Organic Chemistry, University of Chemical Technology and Metallurgy, 1756 Sofia, Bulgaria; pepi_37@abv.bg; 3 Institute of Organic Chemistry with Centre of Phytochemistry, Bulgarian Academy of Sciences, 1113 Sofia, Bulgaria; miroslav.rangelov@orgchm.bas.bg; 4 Institute of Biodiversity and Ecosystem Research, Bulgarian Academy of Sciences, 1113 Sofia, Bulgaria; jane_tch@yahoo.com

**Keywords:** Schiff bases, maximal electroshock seizure, docking, opioid receptors, mice

## Abstract

Four 5,5′-diphenylhydantoin Schiff bases possessing different aromatic species (**SB1**–**SB4**) were recently synthesized and characterized using spectroscopic and electrochemical tools. The present study aimed to ascertain the anticonvulsant activity of the novel phenytoin derivatives **SB1-Ph**, **SB2-Ph**, **SB3-Ph**, and **SB4-Ph**, containing different electron-donor and electron-acceptor groups, and their possible mechanism of action. The **SB2-Ph** exhibited the highest potency to suppress the seizure spread with ED_50_ = 8.29 mg/kg, comparable to phenytoin (ED_50_ = 5.96 mg/kg). While **SB2-Ph** did not produce neurotoxicity and sedation, it decreased locomotion and stereotypy compared to control. When administered in combination, the four Schiff bases decreased the phenytoin ED_50_ by more than 2× and raised the protective index by more than 7× (phenytoin+**SB2-Ph**). The strongest correlation between in-vivo and docking study results was found for ligands’ interaction energies with kappa and delta receptors. These data, combined with the worst interaction energies of our ligands with the mu receptor, suggest that the primary mechanism of their action involves the kappa and delta receptors, where the selectivity to the kappa receptor leads to higher biological effects. Our findings suggest that the four Schiff bases might be promising candidates with potential applications as a safe and effective adjuvant in epilepsy.

## 1. Introduction

Epilepsy is a neurological disorder characterized by spontaneous and unpredictable seizures as its primary symptom. Current treatment strategies in epilepsy involve treating seizures through different approaches, including antiseizure medications (ASMs), resective surgery, and vagus nerve stimulation. However, the biggest challenge in therapy is that approximately 30% of the patients are pharmacoresistant to the applied treatment [1]. Therefore, there is a need to continue the search for novel approaches by inventing a more precise ligand of the targets known for the classical and new-generation ASMs. For modeling and establishing an accurate understanding of protein–ligand interactions, novel antiepileptic drug design relies on shape similarities as the primary descriptors of computational drug discovery.

**Figure 1:**
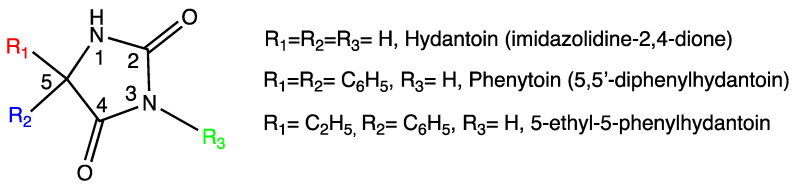
A chemical skeleton of some hydantoin derivatives.

Hydantoin (imidazolidine-2,4-dione) is well-known as a scaffold with a wide range of pharmacological actions [2,3,4]. The discovery of the anticonvulsant properties of 5-ethyl-5-phenylhydantoin and its use as an anti-epileptic drug of choice prompted the synthesis and investigation of many 5,5′-disubstituted hydantoins with varied medical applications (see Figure 1) [5,6]. The most significant hydantoin derivative, used as ASMs against generalized tonic–clonic seizures, is phenytoin (5,5′-diphenylhydantoin) [7]. In experimental protocols, phenytoin exhibited activity in the maximal electroshock seizure (MES) test but failed to suppress seizure activity in other routinely used tests with chemoconvulsants [8]. The literature data suggest the inconsistent bioavailability of phenytoin after oral administration due to its low water and lipid solubility [9,10]. According to Pandeya et al. (2002) [11], the hydrophobic–hydrophilic site of the molecule is critical for the manifestation of the anticonvulsant’s pharmacokinetic effects. Moreover, most of these pharmacophoric units are spaced apart [12,13,14].

Schiff bases are compounds containing imine or azomethine groups (>C=N-), typically synthesized via the condensation of amines and compounds with active carbonyl groups. They are produced from aromatic amines and aldehydes and are known to have numerous applications due to their catalytic activity [15,16,17]. Schiff bases are essential in pharmacy, including as anticancer, anti-inflammatory, and anticonvulsant agents [18]. It has been proposed that the azomethine linkage is responsible for the biological actions of Schiff base derivatives.

Recently, we reported that newly synthesized peptide phenytoin derivatives exhibited anticonvulsant activity in several rodent tests [19]. In addition, the structure–biological activity relationship gave us the grounds to further characterize the novel 5,5′-diphenylhydantoins by incorporating some substituents into the N3 position of the hydantoin ring [20]. Therefore, the objective of the present study was to evaluate the anticonvulsant potency of recently synthesized 3-amino-phenytoin Schiff base derivatives administered alone or in combination with phenytoin in the MES test. Furthermore, a docking analysis was performed to ascertain the role of opioid receptors in their mechanism of action.

## 2. Materials and Methods

### 2.1. The Chemicals and Instrumentation

All reagents and solvents were analytical or high-performance liquid chromatography (HPLC) grade, purchased from Fluka or Merck, and used unpurified. Fourier transform infrared spectroscopy (FTIR), UV-Vis, NMR, high-resolution mass spectrometry (HRMS), electrochemical methods, and single-crystal X-ray diffraction analyses were used to determine the structure of the compounds produced [20].

### 2.2. General Procedure for the Synthesis of 3-Amino-5,5′-diphenylhydantoin Schiff Base Compounds ***SB1-Ph***, ***SB2-Ph***, ***SB3-Ph***, ***SB4-Ph***

All of the phenytoin Schiff bases—(E)-5,5-diphenyl-3-((thiophen-2-ylmethylene)amino)-imidazolidine-2,4-dione (**SB1-Ph**), (E)-3-((2-hydroxybenzylidene)amino)-5,5-diphenylimidazolidine-2,4-dione (**SB2-Ph**), (E)-3-((4-nitrobenzylidene)amino)-5,5-diphenylimidazolidine-2,4-dione (**SB3-Ph**), and (E)-5,5-diphenyl-3-((pyridin-2-ylmethylene)amino)imidazolidine-2,4-dione (**SB4-Ph**)—were prepared by our recently described procedure [20].

### 2.3. Animals and Experimental Design

Adult male ICR mice (23–26 g) were purchased from the vivarium of the Institute of Neurobiology-BAS. They were accommodated in standard Plexiglas cages for a week before experiments in groups of 10 with food and water given ad libitum and were kept in appropriate environmental conditions (artificial 12:12 light–dark cycle with a light on at 7:00 a.m.; temperature of 21 ± 1 °C; humidity: 55 ± 5%). All tests were performed between 9.00 a.m. and 12.00 p.m. The procedures with animals were executed according to the Declaration of Helsinki Guiding Principles on Care and Use of Animals (DHEW Publication, NHI 80-23) and with the EC Directive 2010/63/EU for animal experiments. The project was approved by the Bulgarian Food Safety Agency (License No: 354/2023).

All animals were randomly assigned to experimental groups of six to eight mice per group. The compounds were freshly suspended in 1% dimethyl sulfoxide (DMSO) before each experiment and were administered intraperitoneally (i.p.), in four to six doses for the calculation of ED_50_. Phenytoin was used as a referent drug and administered also i.p. at a time interval of 1 h before testing. In combinations, each compound was applied at a dose of 40 mg/kg 0.5 h before the injection of phenytoin used in four doses to calculate ED_50_. Except for measuring spontaneous activity with the actimeter, each test was conducted 0.5 h after i.p. injection of the vehicle or tested compound.

The mice were assigned to Experiment #1, Experiment #2 and Experiment #3 as follows: Experiment #1: nine groups/four–five doses in the MES test; Experiment #2: ten groups/four doses in the grip strength test and rota-rod test; Experiment #3—six groups/one dose in the actimeter.

### 2.4. MES Test

The test was conducted as described in our previous study [19]. In brief, an electric stimulus of 50 mA, 60 Hz, 0.2 s was applied individually to each tested mouse via corneal electrodes (Constant Current Shock Generator). A criterion for the anticonvulsant activity of treatment was accepted if the hind limb tonic extensor component was suppressed or the animal had clonic seizures.

### 2.5. Grip Strength Test

The mice’s muscle strength was evaluated via the grip strength apparatus attached to the dynamometer (Bioceb, Chaville, France). Each mouse was abruptly pulled backward by the tail after grasping the steel wire grid (8 cm × 8 cm) by their forepaws and the maximal grasping force was assessed until the grid was released by the animal. The grip strength was calculated as the average of three trials and was expressed in N (newtons) ± S.E.M.

### 2.6. Rota-Rod Test

The rota-rod test for neurotoxicity assessment was used as described in a previous study [21]. Impaired motor coordination was considered if the tested animal could not stay without falling from a rotating rod (3.2 cm in diameter, at a speed of 10 rpm) for one minute out of three trials. The dose was considered neurotoxic when more than 50% of the mice failed to keep their balance on the rod.

### 2.7. Measurement of Spontaneous Motor Activity

Two mice were synchronously monitored for motor activity in the actimeter (Infrared Actimeter, Bioseb, France. https://www.bioseb.com/en/activity-motor-control-coordination/51-infrared-actimeter.html, accessed on 16 December 2020). Behavioral data were collected every 10 min for up to 2 h. The animal was set in the apparatus immediately after injection.

### 2.8. Docking of Phenytoin Schiff Bases on Opioid Receptors

Models of the human delta, kappa and mu opioid receptors were constructed and used as docking templates to suggest our ligands’ possible molecular mechanism of action. The XRD structures of the delta, kappa and mu receptors were selected for our modeling based on the resolution and completeness of the deposited data in the Protein Data Bank. The selected PDB codes were 8F7S for the delta receptor, 8F7W for the kappa receptor, and 5C1M for the mu receptor, all of which were stabilized active conformations of human opioid receptors [22].

In all Protein Data Bank structures, minor structural irregularities were corrected followed by the removal of all non-protein species. To achieve the correct protonation state of our receptors, we performed the protonation using the Labute algorithm [23] as implemented in the MOE software package: at pH 7.0, 300 K and a salt concentration of 0.1 m/L, which is the physiological aspect present in the experimental conditions.

In addition, since the subjects in the in-vivo experiments were mice, to mimic the delta, kappa, and mu opioid receptors of mice, the distinctive amino acids of the receptor structures were mutated in silico. For the delta receptor, we used the P32300 Delta-type opioid receptor from the Mus musculus sequence, for the kappa receptor, the P33534 Kappa-type opioid receptor from the Mus musculus sequence, and for the mu opioid receptor, we used the P42866 Mu-type opioid receptor from the Mus musculus; all were from the UniProt database.

We preserved the active region of the receptor during homology modeling without conformational changes, as in the original XRD structures. The respectable reason is that they were crystallized with their active ligands in the cavity, therefore representing the active state of opioid receptors.

All newly synthesized ligands were protonated at pH 7.0 according to their protonation state. The LowModeMD method with the AMBER12 force field was applied to create a conformational library of receptor residues needed for the following docking study. Only conformations with conformational energy up to 5 kcal/mol higher than the lowest energy conformation were used for the next docking.

To find optimal positions for ligand placement in the active site of the receptors during the docking procedure, the Edelsbrunner site-finding algorithm implemented in the MOE software was applied.

The conformations of all ligands were docked into all selected pockets using the Alpha PMI method (MOE2020), as our pockets are quite narrow. The returned poses were evaluated using the London dG function (MOE2020), which estimates the free binding energy of the ligand from a given pose and consists of terms that estimate the average gain/loss of rotational and translational entropy and loss of ligand flexibility, which measure geometric imperfections of hydrogen bonds, and the atomic desolvation energy.

The 100 best positions for each ligand for each pocket were further optimized by the induced fit method using the AMBER12 force field with a generalized Born solvation model and an optimization limit of 6A from the ligand. GBVI/WSA dG (MOE2020) was used as a scoring function, and the best 30 poses were collected for the subsequent analysis.

### 2.9. Statistical Analysis

The dose of each compound that produced the desired endpoint in 50% of mice (ED_50_ or TD_50_) in the MES and rotarod test, respectively, and a 95% confidence interval were evaluated by the computer-assisted log-probit analysis described by Finney (1971) [24]. The protective index (PI) was calculated as a ratio of TD_50_/ED_50_. Data from the grip-strength test and spontaneous motor activity were verified with one-way ANOVA. In the case of significant differences, a post hoc test was used. Results were considered statistically significant if *p* < 0.05.

## 3. Results

### 3.1. Chemistry

**Scheme 1:**
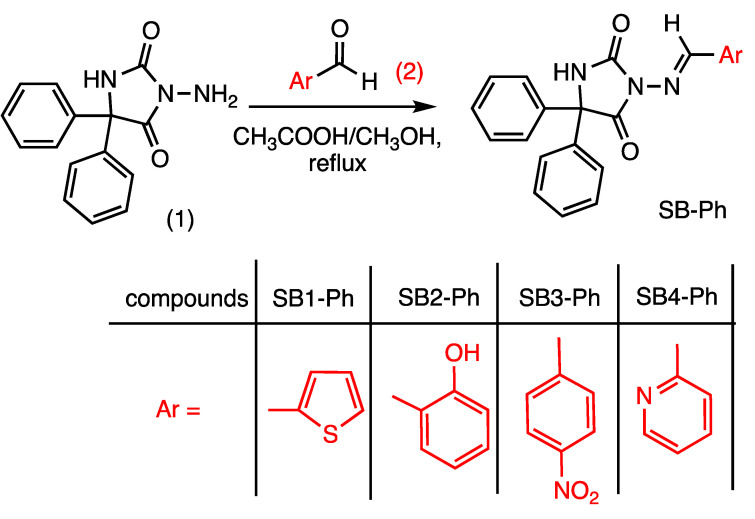
Synthesis of 3-amino-5,5′-diphenylhydantoin Schiff bases.

Four new 3-amino-5,5′-diphenylhydantoin Schiff Bases (**SB1-Ph**, **SB2-Ph**, **SB3-Ph**, **SB4-Ph**) were synthesized as described in detail [20]. The studied phenytoin compounds are presented in Table 1 and referred to by their designation in [20]. The novel phenytoin Schiff bases were synthesized by a condensation reaction in absolute methanol between 3-amino-5,5′-diphenylimidazolidine-2,4-dione (1) and the corresponding aromatic aldehyde (2) in a 1:1 molar ratio in the presence of catalytic quantities of glacial acetic acid (Scheme 1).

**SB1-Ph** and **SB4-Ph** have a donor thiophene/pyridine ring, but **SB3-Ph** has an acceptor 4-nitrophenyl moiety. **SB2-Ph** has a 2-hydroxyphenyl portion with an intramolecular hydrogen bonding between the phenol -OH group and the azomethine nitrogen nonbonding electron pair.

Table 1 shows the abbreviation, molecular formula, and molecular weight of investigation phenytoin compounds as follows: (E)-5,5-diphenyl-3-((thiophen-2-ylmethylene)amino)imidazolidine-2,4-dione (**SB1-Ph**), (E)-3-((2-hydroxybenzylidene)amino)-5,5-diphenylimidazolidine-2,4-dione (**SB2-Ph**), (E)-3-((4-nitrobenzylidene)amino)-5,5-diphenylimidazolidine-2,4-dione (**SB3-Ph**), and (E)-5,5-diphenyl-3-((pyridin-2-ylmethylene)amino)imidazolidine-2,4-dione (**SB4-Ph**).

### 3.2. Anticonvulsant Activity

The phenytoin Schiff bases, administered alone, exhibited an anticonvulsant effect in the MES test, and their ED_50_ value are shown in Table 2. The order of potency for the four compounds was as follows: **SB2-Ph** > **SB4-Ph** > **SB3-Ph** > **SB1-Ph**. When the phenytoin Schiff bases were used in combination with phenytoin at a fixed dose of 40 mg/kg, they decreased the ED*_50_* of the ASM as follows: 2× (**SB1-Ph**), 1.9× (**SB2-Ph**), 2.23× (**SB3-Ph**) and 3.37 (**SB4-Ph**), respectively. In addition, the combinations were characterized by an elevation of the PI as follows: 6.23 (**SB1-Ph**), 7.8× (**SB2-Ph**), 3.86× (**SB3-Ph**) and 7.19 (**SB4-Ph**), respectively.

In the rota-rod test, the four novel phenytoin derivates exhibited a higher TD_50_ than the referent ASM, with almost two times higher TD_50_ for **SB3-Ph** than phenytoin (**SB1-Ph**—1.6×; **SB2-Ph**—1.47×; **SB3-Ph**—1.77; **SB4-Ph**—1.14) (Table 1), suggesting low potential neurotoxicity. Furthermore, when the four phenytoin Schiff bases were administered in combination with different doses of phenytoin, the TD_50_ was about 3× for phenytoin+**SB1-Ph**; >4× for phenytoin+**SB2-Ph**; 1.7× for phenytoin+**SB3-Ph** and 2.14 for phenytoin+**SB4-Ph**.

### 3.3. Muscle Strength and Spontaneous Motor Activity

#### 3.3.1. Muscle Strength

The novel phenytoin Schiff bases, when administered alone at a dose of 40 mg/kg, or used in combination with phenytoin, did not change the muscle strength measured by the grip strength apparatus (Table 3). Similarly, this parameter was not affected when the compounds were treated in combination with phenytoin at a dose given alone, suggesting the lack of a sedative effect.

#### 3.3.2. Spontaneous Motor Activity

**Figure 2:**
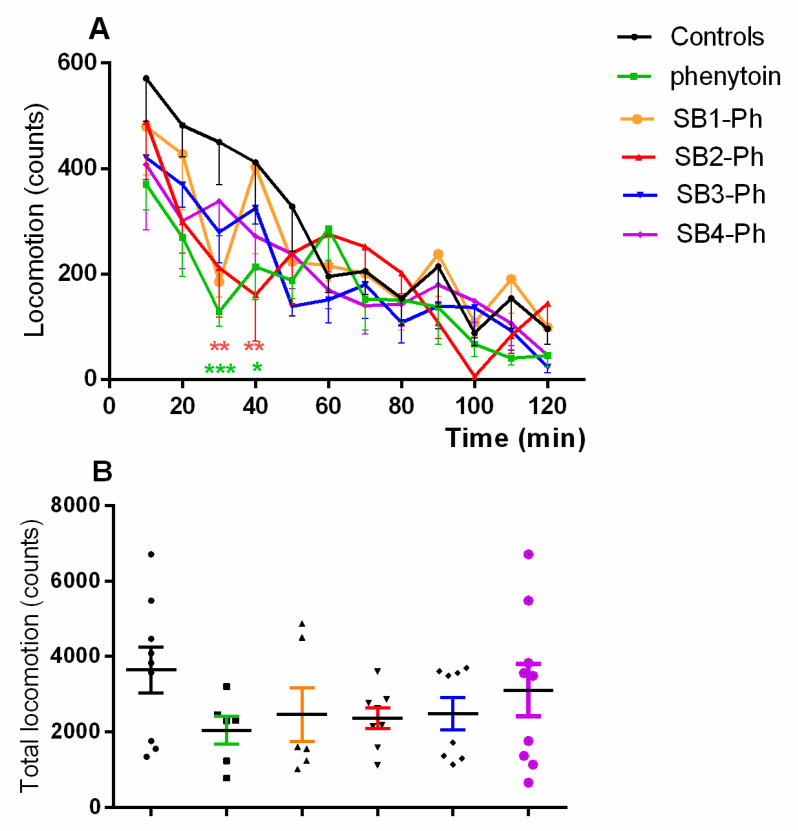
Locomotion (**A**) and Total activity (**B**) were registered for two hours in the Actimeter in mice injected immediately before registration with a vehicle, phenytoin, **SB1-Ph**, **SB2-Ph**, **SB3-Ph**, **SB4-Ph**. Data are presented as mean ± SEM, *n* = 6/group. *** *p* < 0.001 and * *p* = 0.022, phenytoin compared to controls; ** *p* = 0.006 and ** *p* = 0.004, **SB2-Ph** compared to controls (**A**).

**Figure 3:**
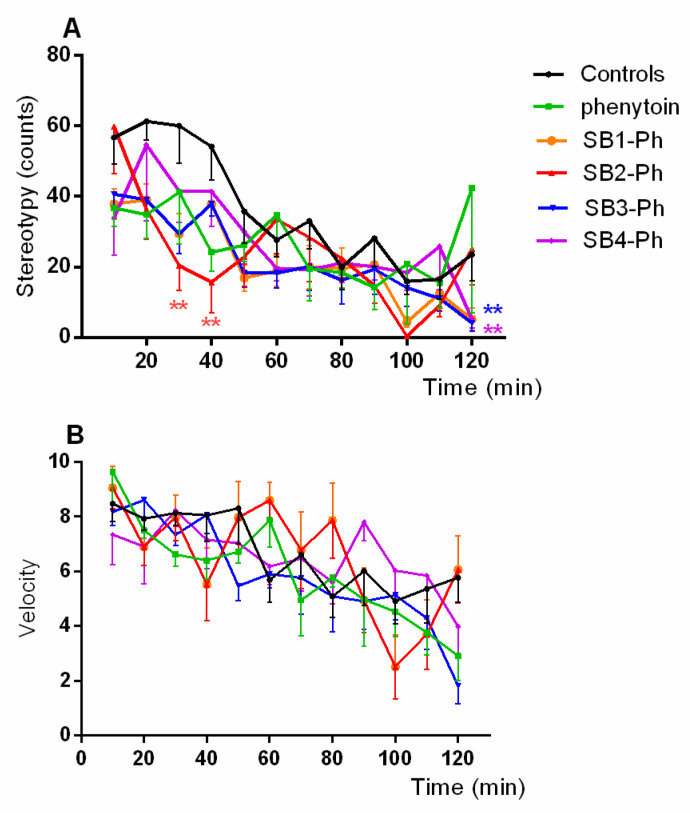
Stereotypy (**A**) and Velocity (**B**) were registered for two hours in the Actimeter in mice injected immediately before registration with a vehicle, phenytoin, **SB1-Ph**, **SB2-Ph**, **SB3-Ph**, **SB4-Ph**. Data are presented as mean ± SEM, *n* = 6/group. ** *p* = 0.005, **SB2-Ph** compared to controls; ** *p* = 0.005, **SB3-Ph** compared to controls; ** *p* = 0.006, **S4-Ph** compared to controls (**A**).

The spontaneous activity of the phenytoin analogs and the referent drug phenytoin was registered for 2 h in the actimeter immediately after their i.p. administration. A main time [two-way ANOVA: F_11,455_ = 16.893, *p* < 0.001] for spontaneous activity and stereotypy [F_11,455_ = 7.33, *p* < 0.001] suggested that phenytoin analogs exhibited habituation with a tendency for decreased activity in time. Furthermore, a main drug effect was demonstrated for locomotion [F_4,455_ = 6.093, *p* < 0.001] and stereotypy [F_4,455_ = 6.136, *p* < 0.001]. Post hoc analysis revealed that, similarly to the referent drug phenytoin, the **SB2-Ph** compound had a decreased motor activity detected at the 30th and 40th minutes compared to the control group (phenytoin vs. control, 30th min: *p* < 0.001, 40th min: *p* = 0.022; **SB2-Ph** vs. control, 30th min: *p* = 0.006, 40th min: *p* = 0.004) (Figure 2A). No significant difference among control and groups treated with the highest dose of 40 mg/kg was detected for the total locomotion (*p* > 0.05) (Figure 2B). For stereotypy, a significant decrease compared to the control group of this parameter was detected for **SB2-Ph** at the 30th min: *p* = 0.005; 40th min: *p* = 0.005, **SB3-Ph** vs. control at the 120th min: *p* = 0.005; and **SB4-Ph** vs. control at the 120th min: *p* = 0.006 (Figure 3A). As for total motor activity, no difference among control and groups treated with the highest dose of 40 mg/kg was detected for the velocity of movement (*p* > 0.05) (Figure 3B).

### 3.4. Docking Analysis

All three receptors have active sites with a shape and volume capable of hosting our ligands. All three pockets are mostly lipophilic, with nearly the same amount of hydrophilic amino acids. The three receptor molecules contain more basic than acidic amino acids in their pockets, distributed in all parts of the pockets.

**Figure 4:**
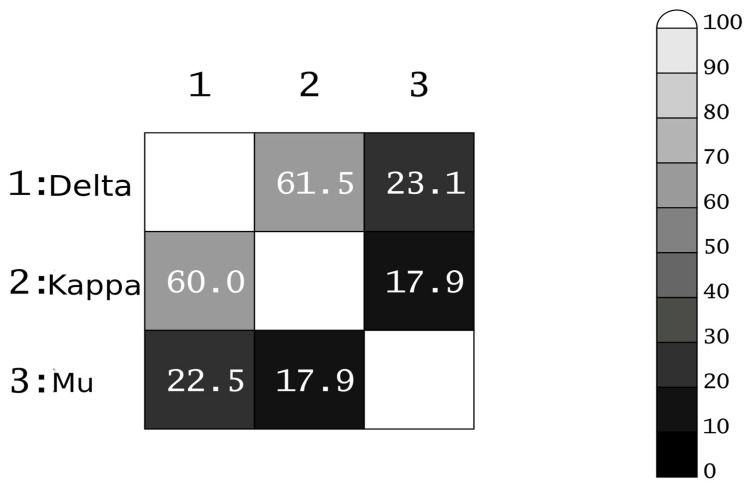
The similarity between amino acids forming active sites of our delta, kappa and mu opioid receptor models.

Mu opioid receptors do not have acidic residues in the active site. In contrast, kappa and delta receptors have amino acids positioned in the upper part of the pocket. Most of them are strongly exposed to the solvent and interact mainly with solvent molecules. As shown in Figure 4, pockets of kappa and delta receptors are much more similar to each other than kappa or delta are to mu receptors (Figure 4).

**Figure 5:**
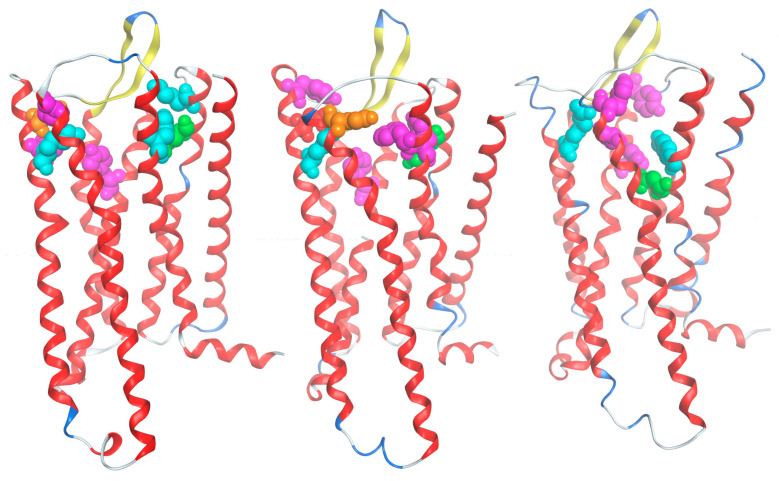
Interactions between receptors and ligands at their best poses presented by maps depicting polar and lipophilic amino acids.

We prepared interaction maps depicting polar amino acids in pink, while lipophilic ones are in green to present interactions between receptors and our ligands in their best poses (Figure 5). Acidic amino acids are circled with red, while the basic ones are circled with blue. Side-chain interactions are depicted with green arrows. The interaction with the backbone is in blue, where the arrowhead points to the hydrogen bond acceptor. Exposure to the solvent is depicted with a blue halo around the ligand atoms and blue circles around the amino acids of the receptor. A gray dotted line shows the proximity contour of the pocket.

**Figure 6:**
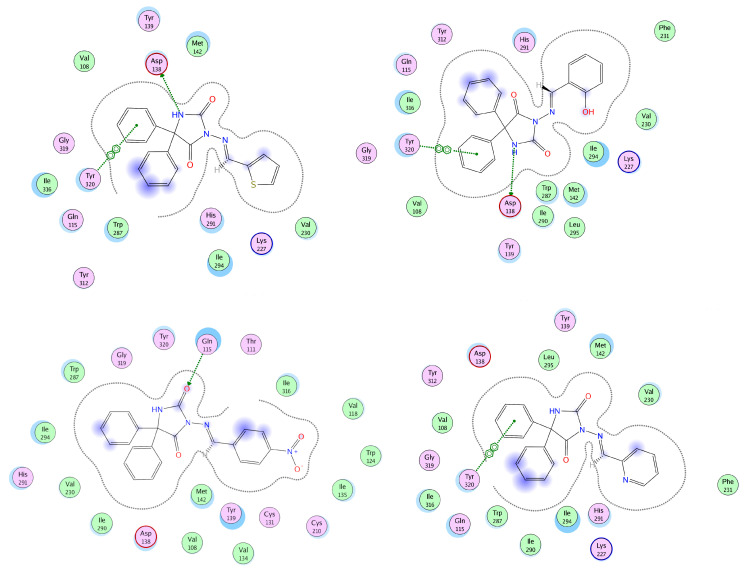
Interaction maps of our ligands’ best poses with kappa opioid receptor model. **SB1-Ph** upper left, **SB2-Ph** upper right, SB3-Ph lower left, **SB4-Ph** lower right.

Our ligand that best interacts with the kappa receptor is **SB3-Ph**, where one of the carbonyl oxygens forms a hydrogen bond with Gln115 and the nitro group is near Cys210 (Figure 6).

**Figure 7:**
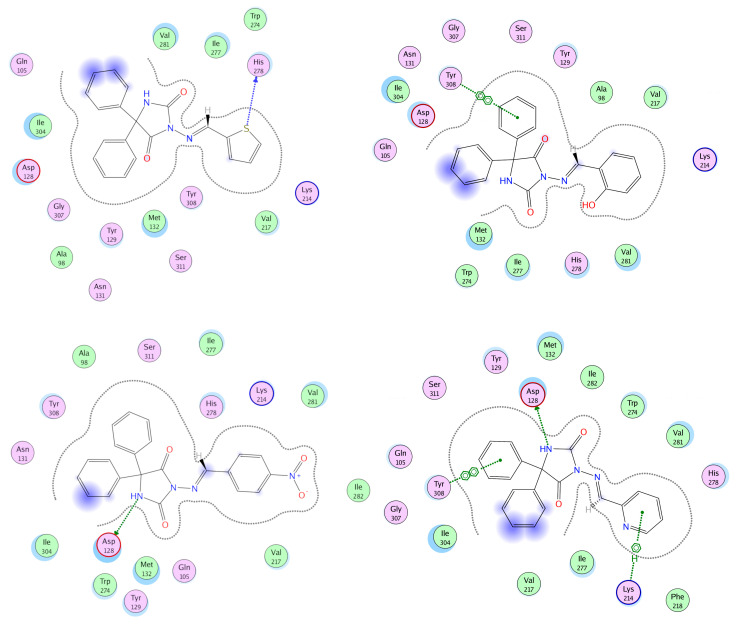
Interaction maps of the best poses of our ligands with the model of delta opioid receptor. **SB1-Ph** upper left, **SB2-Ph** upper right, SB3-Ph lower left, **SB4-Ph** lower right.

In the case of the delta receptor, the best ligands are **SB2-Ph** and **SB3-Ph**, which have roughly the same interaction energies and fit well in the receptor cavity (Figure 7). **SB3-Ph** forms a hydrogen bond with Asp128, which, in contrast to **SB2-Ph**, will lead to greater selectivity for its positioning inside the receptor interior. The worst performing ligand is, as in the case of kappa receptor, **SB1-Ph**. All ligands prefer to interact relatively in the same space and orientation inside the delta receptor active site cavity.

**Figure 8:**
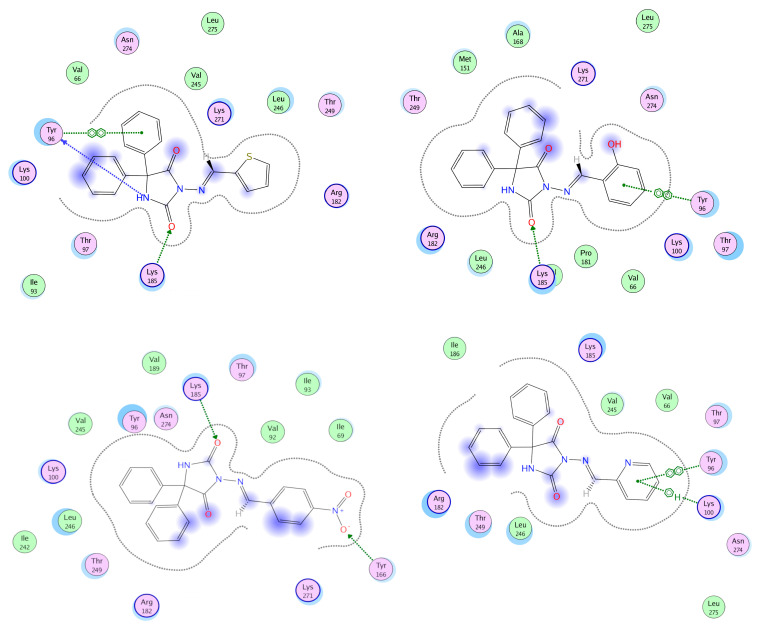
Interaction maps of our ligands’ best poses with the mu opioid receptor model. **SB1-Ph** upper left, **SB2-Ph** upper right, **SB3-Ph** lower left, **SB4-Ph** lower right.

In the case of the mu opioid receptor, the best ligand is also **SB3-Ph**, which fits firmly in the receptor active site interior and forms two hydrogen bonds: one between the nitro group and Tyr166 and another between Lys185 and the carbonyl oxygen group (Figure 8). The last hydrogen bond is also formed in ligands **SB1-Ph** and **SB2-Ph**. The worst interaction with the pocket has **SB4-Ph**, which did not form any hydrogen bond with the pocket amino acids.

**Figure 9:**
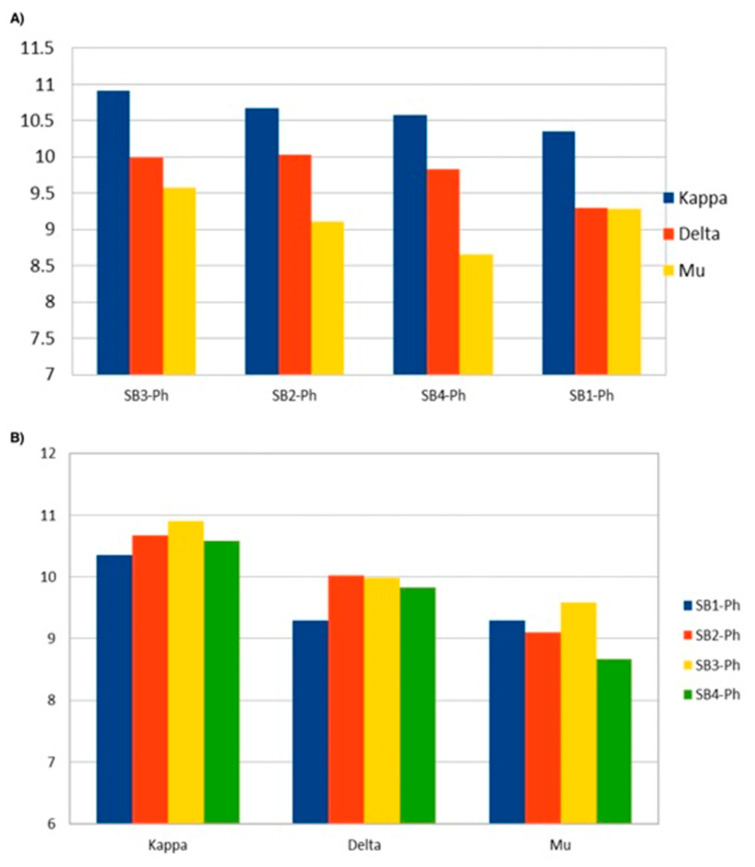
Interaction energies of the best poses of our ligands with kappa, delta and mu opioid receptor (**A**). Interaction energies of the best poses of **SB1-Ph**, **SB2-Ph**, **SB3-Ph** and **SB4-Ph** with kappa, delta and mu opioid receptor (**B**).

In all cases (delta, kappa and mu receptors), **SB3-Ph** is the ligand with the highest interaction energy (in the case of the delta receptor, **SB3-Ph** and **SB2-Ph** interact equally strongly (Figure 9)).

**Figure 10:**
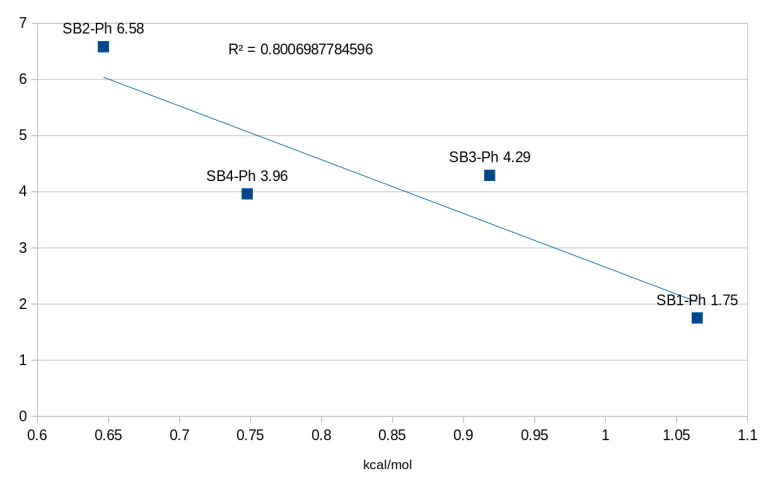
Correlation of difference between interaction energies of our ligands with kappa and delta receptors vs. experimental data of their protective index (PI).

We have tried different correlations between experimental data and results from the docking study and found that the experiment best correlates with the difference between the interaction energies of ligands with kappa and delta receptors, where R^2^ is 0.8 (Figure 10).

## 4. Discussion

The phenytoin Schiff bases (**SB1-Ph**, **SB2-Ph**, **SB3-Ph**, **SB4-Ph**), synthesized from 3-amino-5,5′-diphenylimidazolidine-2,4-dione and a corresponding aromatic aldehyde, were recently characterized in detail using X-ray, optical and electrochemical methods [20]. Still, no study on pharmacology has been conducted. Phenytoin was the first ASM showing anticonvulsant efficacy in the MES test before being approved for human treatment [25]. Nowadays, corneal stimulation in mice is still the first choice for screening and is accepted as the “gold standard” for discovering new drugs with potential efficacy against tonic–clonic seizures [26]. The mechanism of anticonvulsant action of phenytoin, associated with the suppression of voltage-gated sodium channels, explains its specific activity against the MES test and ability to abolish the seizure spread [8,27]. The ED_50_ value of 5.96 mg/kg for phenytoin in the present study is close to that previously reported [27,28].

In the present study, we applied an MES test to evaluate the anticonvulsant effect of the four novel phenytoin Schiff bases and to characterize their potential to amplify the activity of the referent drug, phenytoin, without leading to adverse side effects. The combination of phenytoin with the phenytoin Schiff bases was additive in the MES test and is expected to produce hyper-additive outcomes in clinical practice. The significant increase in the protective index (more than seven times in the case with **SB2-Ph**) demonstrated the advantage of the combination in increasing the safety of this type of a treatment mode.

Overall, the results from the MES test revealed that **SB2-Ph** is the most active compound when administered alone, suggesting the crucial role of a 2-hydroxyphenyl portion insertion to the 3-amino-5,5′-diphenylimidazolidine-2,4-dione for the anticonvulsant potency of this compound. The Schiff base **SB2-Ph** had a higher ED_50_ value than the referent drug (**SB2-Ph**: ED_50_ = 8.29 mg/kg vs. phenytoin: ED_50_ = 5.96 mg/kg). The PI value represents a TD_50_/ED_50_ ratio, and this parameter determines the safety level of the tested compound. The present results showed that the PI = 6.58 of **SB2-Ph** is superior to the other tested novel Schiff bases and comparable to the PI of 6.22 calculated experimentally for the referent drug, phenytoin. Further, in combined treatment (phenytoin+**SB4-Ph**), the pyridine ring donor to 3-amino-5,5′-diphenylimidazolidine-2,4-dione seems to exert the highest amplification of the phenytoin anticonvulsant activity, decreasing the ED_50_ about 3× and elevating PI 7×. The other three phenytoin Schiff bases (**SB1-Ph**, **SB2-Ph and SB3-Ph**) also reduced the ED_50_ of phenytoin about 2× and raised the PI from 4× to 8×. Based on this experimental study, one can conclude that combining a phenytoin Schiff base with phenytoin could significantly diminish the ED_50_ of this classical ASM and amplify its PI while attenuating possible adverse effects. However, although **SB2-Ph** did not produce neurotoxicity and sedation, it decreased locomotion and stereotypy compared to the control group 30 and 40 min after injection.

The findings from the docking analysis in the present study suggest that the underlying mechanism of the anticonvulsant activity of the four 5,5′-diphenylhydantoin Schiff bases, which are structurally similar to phenytoin, is different. Thus, whereas the primary molecular mechanism of phenytoin involves a blockade of the fast voltage-gated Na^+^ channels responsible for action potential [8,27], the kappa and delta opioid receptors might be involved in the action of newly synthesized analogs, suggesting that the Schiff base could be the critical pharmacophore.

Experimental results and data from patients with epilepsy demonstrated the reduced function of the opioid system in brain regions vulnerable to epileptiform activity and with low seizure thresholds (reviewed in: [29]). The literature data showed that activating the kappa opioid receptor is necessary for the anticonvulsant action of endogenous dynorphin, suggesting that these receptors have neuroprotective functions. On the other hand, the findings related to delta opioid receptors revealed that they play a dual role in seizure susceptibility. Nevertheless, the crucial impact of the opioid system in brain regions vulnerable to epileptogenesis stimulates the research on the design and discovery of promising molecule ligands on kappa/delta receptors.

The best correlation between experimental in-vivo data and docking study results was found for ligands’ interaction energies with kappa and delta opioid receptors. This result, combined with the worst interaction energies of our ligands with mu opioid receptors, leads to the conclusion that kappa and delta opioid receptors are included in the primary mechanism of action of our ligands, where higher selectivity to kappa receptor leads to a more substantial biological effect. The primary term in the energy is favorable lipophilic interactions and the lack of significant sterical hindrances. Lipophilic interactions are proposed to play an essential role in the affinity of the natural ligands for the kappa opioid receptor [30].

Gln115 is an essential part of the subpocket in the active site, where some potent specific agonists bind, and its mutation reduces the agonist activity [31]. Cys210, situated in ECL2, is one of the most conserved residues among delta, kappa, and mu opioid receptors, supposed to play an important role in the kappa receptor ligand’s affinity [30]. Due to the lack of a polar p-nitro group, other ligands are shorter and tend to take a more horizontal position toward the internal cavity of the receptor. **SB1-Ph** and **SB2-Ph** prefer to orient the polar part from their variable fragment of the structure toward Lys227, of the three key interactions responsible for the activation of the kappa receptor [32], while **SB2-Ph** forms an intramolecular hydrogen bond between the phenolic group and the near azo N atom. Notably, Lys 227 change by a mutational analysis reports an affected receptor function rather than a direct effect on the ligand.

**SB1-Ph** is the most unfavorable ligand due to the sterical hindrance of the giant S atom in the variable ring part of the ligand molecule. For kappa opioid receptors, a detailed analysis of the best 50 poses of our ligands shows that the most frequent interaction between our ligands and the active site includes Gln115, as in the case of our best-interacting ligand **SB3-Ph**. The second one in frequency of interaction is Asp138, which is conserved in all opioid receptors and plays a critical role in the kappa receptor binding pocket and activation [30], as it participates in an ionic interaction with ligands [33]. It is known from analyzed selective kappa receptor agonists that Tyr320 is involved in interactions with ligands as hydrophobic residue [34], which can be seen in cases of our ligands, too. Tyr320 is also a key determinant for ligand activity and receptor activation, proving its mutation significantly reduces or eliminates further signal transduction [31].

For the delta opioid receptor, a detailed analysis of the best 50 poses of our ligands shows that the most frequent interaction between our ligands and the active site includes Asp128, as in the case of our best-interacting ligand **SB3-Ph**. Asp128 and its ability to form an H-bond with the ligand is mentioned, and it is recognized as a critical residue in the study of Collu2012. Asp128 and Tyr308, which interact with some of the best poses of our ligands, are mentioned by Wang et al. (2023) [22] for being part of the N-terminal natural ligand recognition region Y[D-Ala]F, similar to the tetrade in the mu opioid receptor. Still, they are not among the lipophilic amino acids, proven as necessary for specificity by mutational analysis [35,36,37].

For the mu opioid receptor, a detailed analysis of the best 50 poses of our ligands shows that the most frequent interaction between them and the active site includes Lys185 as a hydrogen bond acceptor, as in the case of our best-interacting ligand **SB3-Ph**. The interaction of our best ligand with Tyr166 is interesting, as Tyr166, besides being highly conserved, when phosphorylated, may reduce the mu opioid receptor–G-protein coupling efficiency and thus agonist efficiency [38], which is prevented when it is blocked in the interaction with our ligand. The mutation of the other Tyr96, which interacts with our ligands, did not affect signaling.

## 5. Conclusions

The present study revealed that four 3-amino-5,5′-diphenylhydantoin Schiff bases (**SB1-Ph, SB2-Ph, SB3-Ph, SB4-Ph**) recently synthesized from our team possess anticonvulsant activity in the MES test in mice at doses that did not produce adverse side effects. The insertion of a 2-hydroxyphenyl to the 3-amino-5,5′-diphenylimidazolidine-2,4-dione scaffold was comparable to the ASM phenytoin activity in the **SB2-Ph** compound. Combining the four phenytoin analogs in a fixed dose producing no sedation with phenytoin led to a substantial ED_50_ reduction of this ASM and an elevation of PI, which might be expected to be a clinically desirable outcome.

The best correlation between experimental in-vivo data and docking study results was found for ligands’ interaction energies with kappa and delta opioid receptors. This finding, combined with the worst interaction energies of our ligands with the **mu** opioid receptors, leads to the conclusion that kappa and delta opioid receptors are included in the primary mechanism of action of our ligands, where higher selectivity to kappa receptors leads to a more substantial biological effect. Experimental results suggest that these four novel Schiff bases could be potential adjuvant candidates for developing safe and effective drugs for epilepsy.

## Figures and Tables

**Table 1:** Some chemical parameters of the phenytoin Schiff bases.

Abbreviation	Compound	Molecular Formula	Molecular Weight
**SB1-Ph**	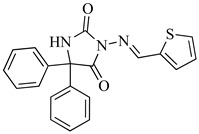	C_20_H_15_N_3_O_2_S	362.0885
**SB2-Ph**	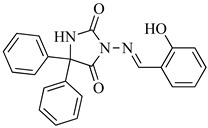	C_22_H_17_N_3_O_3_	372.1270
**SB3-Ph**	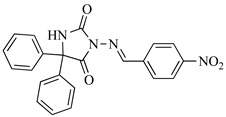	C_22_H_16_N_4_O_4_	401.1172
**SB4-Ph**	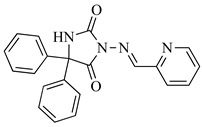	C_21_H_16_N_4_O_2_	357.1273

**Table 2:** Quantitative assessment of anticonvulsant activity of phenytoin Schiff bases and their combination with different doses of phenytoin in the MES and rotarod test in mice.

Drug	^a^ TPE (min)	^b^ ED_50_ mg·kg^−1^	95% Confidence Interval	^c^ TD_50_	^d^ PI
phenytoin	60	5.96	(4.65–7.64)	37.07	6.22
**SB1-Ph**	30	34.09	(17.66–65.79)	59.55	1.75
phenytoin+**SB1-Ph**	60 + 30	2.91	(1.87–4.53)	112.8	38.76
**SB2-Ph**	30	8.29	(5.58–12.33)	54.51	6.58
phenytoin+SB2-Ph	60 + 30	3.10	(1.20–8.02)	>150	48.39
**SB3-Ph**	30	15.29	(6.73–34.75)	65.62	4.29
phenytoin+**SB3-Ph**	60 + 30	2.67	(1.24–5.72)	64.13	24.02
**SB4-Ph**	30	10.71	(5.92–19.38)	42.37	3.96
phenytoin+**SB4-Ph**	60 + 30	1.77	(0.97–3.2)	79.20	44.75

^a^ Time to peak effect—TPE; ^b^ median effective doses (ED_50_); ^c^ median minimal neurotoxic doses (TD_50_) and ^d^ protective index (PI) (rota-rod TD_50_/ED_50_).

**Table 3:** Effects of new modified analogs of phenytoin—**SB1-Ph**, **SB2-Ph**, **SB3-Ph** and **SB4-Ph** and **combinations** of phenytoin**+SB1-Ph**; **phenytoin+SB2-Ph**; **phenytoin+SB3-Ph** and **phenytoin+SB4-Ph** on neuromuscular tone in the grip-strength test and motor coordination in the rotarod test in mice.

Group/Treatment	Dose (mg/kg). i.p.	Neuromuscular Strength (N)	Rotarod Test N/F
Control (saline)	0	2.08 ± 0.48	0/8
phenytoin	2.5	1.98 ± 0.13	1/6
	5	2.20 ± 0.38	0/6
	10	1.84 ± 0.18	2/6
	20	2.06 ± 0.49	0/6
**SB1-Ph**	5	2.25 ± 0.35	1/6
	10	2.07 ± 0.67	2/6
	20	1.82 ± 0.36	2/6
	40	2.52 ± 0.32	1/6
phenytoin+**SB1-Ph**	2.5 + 10	2.22 ± 0.33	1/6
	5 + 10	2.38 ± 0.34	1/6
	10 + 10	2.38 ± 0.21	0/6
	20 + 10	1.51 ± 0.22	0/6
**SB2-Ph**	2.5	2.08 ± 0.34	1/6
	5	2.39 ± 0.35	1/6
	10	1.56 ± 0.28	1/6
	20	1.62 ± 0.39	2/6
	40	1.74 ± 0.29	2/6
phenytoin+**SB2-Ph**	3.5 + 10	1.54 ± 0.21	0/6
	5 + 10	1.55 ± 0.25	0/6
	10 + 10	1.97 ± 0.39	0/6
	20 + 10	1.70 ± 0.19	0/6
**SB3-Ph**	5	1.91 ± 0.28	1/6
	10	1.98 ± 0.53	2/6
	20	1.85 ± 0.39	2/6
	40	1.69 ± 0.30	0/6
phenytoin+**SB3-Ph**	2.5 + 10	2.27 ± 0.39	2/6
	5 + 10	2.27 ± 0.38	0/6
	10 + 10	1.76 ± 0.44	1/6
	20 + 10	2.22 ± 0.23	1/6
**SB4-Ph**	1	2.06 ± 0.28	0/6
	5	2.18 ± 0.43	0/6
	10	2.0 ± 0.41	0/6
	20	1.94 ± 0.4	1/6
	40	1.93 ± 0.32	3/6
phenytoin+**SB4-Ph**	2.5 + 10	1.89 ± 0.26	1/6
	5 + 10	2.15 ± 0.27	2/6
	10 + 10	2.14 ± 0.23	0/6
	20 + 10	2.09 ± 0.38	2/6

Data are presented as mean muscle strength (in Newtons ± S.D. of 3 determinations) in mice subjected to the grip-strength test and number protected vs. all tested mice from the rotarod test. Positive mean reached the criterion (see Section 2). N/F = number of animals toxic over the number tested; The analogs were injected i.p. 15 min before the tests at different doses and combinations as shown above.

## References

[1] Remy S., Beck H. (2006). Molecular and cellular mechanisms of pharmacoresistance in epilepsy. Brain.

[2] Alanazi A.M., El-Azab A.S., Al-Swaidan I.A., Maarouf A.R., El-Bendary E.R., Abu El-Enin M.A., Abdel-Aziz A.A.-M. (2013). Synthesis, single-crystal, in vitro antitumor evaluation and molecular docking of 3-substitued 5,5-diphenylimidazolidine-2,4-dione derivatives. Med. Chem. Res..

[3] Alaa A.M., El-Azab A.S., Abou-Zeid L.A., ElTahir K.E.H., Abdel-Aziz N.I., Ayyad R.R., Al-Obaid A.M. (2016). Synthesis, anticancer, apoptosis-inducing activities and EGFR and VEGFR2 assay mechanistic studies of 5,5-diphenylimidazolidine-2,4-dione derivatives: Molecular docking studies. Eur. J. Med. Chem..

[4] Zhang M., Liang Y.R., Li H., Liu M.M., Wang Y. (2017). Design, synthesis, and biological evaluation of hydantoin bridged analogues of combretastatin A-4 as potential anticancer agents. Bioorg. Med. Chem..

[5] Alkahtani H.M., Alanazi M.M., Aleanizy F.S., Alqahtani F.Y., Alhoshani A., Alanazi F.E., Almehizia A.A., Abdalla A.N., Alanazi M.G., El-Azab A.S. (2019). Synthesis, anticancer, apoptosis-inducing activities and EGFR and VEGFR2 assay mechanistic studies of 5, 5-diphenylimidazolidine-2, 4-dione derivatives: Molecular docking studies. Saudi Pharm. J..

[6] Brown M.L., Brown G., Brouillette W.J. (1997). Effects of log P and phenyl ring conformation on the binding of 5-phenylhydantoins to the voltage-dependent sodium channel. J. Med. Chem..

[7] Yaari Y., Selzer M.E., Pincus J.H. (1986). Phenytoin: Mechanisms of its anticonvulsant action. Ann. Neurol..

[8] Krall R., Penry J.K., White B.G., Kupferberg H.J., Swinyard E.A. (1978). Antiepileptic Drug Development: II. Anticonvulsant Drug Screening. Epilepsia.

[9] Varia S.A., Stella V.J. (1984). Phenytoin Prodrugs V: In Vivo Evaluation of Some Water-Soluble Phenytoin Prodrugs in Dogs. J. Pharm. Sci..

[10] Suzuki T., Saitoh Y., Nishihara K. (1970). Kinetics of Diphenylhydantoin Disposition in Man. Chem. Pharm. Bull..

[11] Pandeya S.N., Raja A.S., Stables J.P. (2002). Synthesis of isatin semicarbazones as novel anticonvulsants—Role of hydrogen bonding. J. Pharm. Pharm. Sci..

[12] Alachkar A., Ojha S., Sadeq A., Adem A., Frank A., Stark H., Sadek B. (2020). Experimental models for the discovery of novel anticonvulsant drugs: Focus on pentylenetetrazole-induced seizures and associated memory deficits. Curr. Pharm. Des..

[13] Jain J., Kumar Y., Stables J., Sinha R. (2010). Menthone semicarbazides and thiosemicarbazides as anticonvulsant agents. Med. Chem..

[14] Unverferth K., Engel J., Höfgen N., Rostock A., Günther R., Lankau H.J., Menzer M., Rolfs A., Liebscher J., Muller B. (1998). Synthesis, Anticonvulsant Activity, and Structure−Activity Relationships of Sodium Channel Blocking 3-Aminopyrroles. J. Med. Chem..

[15] Patai S. (1970). The Chemistry of the Carbon-Nitrogen Double Bond.

[16] Prakash A., Adhikari D. (2011). Application of Schiff Bases and Their Metal Complexes—A Review. Int. J. ChemTech. Res..

[17] Weng Q., Yi J., Chen X., Luo D., Wang Y., Sun W., Kang J., Han Z. (2020). Controllable Synthesis and Biological Application of Schiff Bases from d-Glucosamine and Terephthalaldehyde. ACS Omega.

[18] Cates A.L., Rasheed S.M. (1984). Phosphorus GABA Analogues as Potential Prodrugs. Pharm. Res..

[19] Todorov P., Peneva P., Georgieva S., Tchekalarova J., Rangelov M., Todorova N. (2022). Synthesis and characterization of new 5,5’-dimethyl- and 5,5’-diphenylhydantoin-conjugated hemorphin derivatives designed as potential anticonvulsant agents. New J. Chem..

[20] Todorov P., Georgieva S., Peneva P., Rusew R., Shivachev B., Georgiev A. (2020). Experimental and theoretical study of bidirectional photoswitching behavior of the 5,5’-diphenylhydantoin Schiff bases: Synthesis, crystal structure and kinetics approaches. New J. Chem..

[21] Sills G.J., Brodie M.J., Schwartzkroin P.A. (2009). Encyclopedia of Basic Epilepsy Research.

[22] Wang Y., Zhuang Y., Di Berto J.F., Zhou X.E., Schmitz G.P., Yuan Q., Jain M.K., Liu W., Melcher K., Jiang Y. (2023). Structures of the entire human opioid receptor family. Cell.

[23] Labute P. (2008). The generalized Born/volume integral implicit solvent model: Estimation of the free energy of hydration using London dispersion instead of atomic surface area. J. Comput. Chem..

[24] Finney D.J. (1971). Probit Analysis.

[25] Putnam T.J., Merritt H. (1937). Experimental determination of the anticonvulsant properties of some phenyl derivatives. Science.

[26] Bialer M., White H.S. (2010). Key factors in the discovery and development of new antiepileptic drugs. Nat. Rev. Drug Discov..

[27] Löscher W. (2016). Fit for purpose application of currently existing animal models in the discovery of novel epilepsy therapies. Epilepsy Res..

[28] Barton M.E., Klein B.D., Wolf H.H., White H.S. (2001). Pharmacological characterization of the 6 Hz psychomotor seizure model of partial epilepsy. Epilepsy Res..

[29] Burtscher J., Schwarzer C. (2017). The Opioid system in temporal lobe epilepsy: Functional role and therapeutic potential. Front. Mol. Neurosci..

[30] Vardy E., Mosier P.D., Frankowski K.J., Wu H., Katritch V., Westkaemper R., Aubé J., Stevens R.C., Roth B.L. (2013). Chemotype-selective modes of action of κ-opioid receptor agonists. J. Biol. Chem..

[31] Han J., Zhang J., Nazarova A.L. (2023). Ligand and G-protein selectivity in the κ-opioid receptor. Nature.

[32] Jonas H., Aiello D., Schepmann D., Diana P., Wünsch B. (2022). Synthesis of 8-aminomorphans with high KOR affinity. Eur. J. Med. Chem..

[33] Wu H., Wacker D., Mileni M., Katritch V., Han G.W., Vardy E., Liu W., Thompson A.A., Huang X.P., Carroll F.I. (2012). Structure of the human κ-opioid receptor in complex with JDTic. Nature.

[34] Singh N., Cheve G., Ferguson D., McCurdy C.R. (2006). A combined ligand-based and target-based drug design approach for G-protein coupled receptors: Application to salvinorin A, a selective kappa opioid receptor agonist. J. Comput. Aided Mol. Des..

[35] Fadhil I., Schmidt R., Walpole C., Carpenter K.A. (2004). Exploring deltorphin II binding to the third extracellular loop of the delta-opioid receptor. J. Biol. Chem..

[36] Fenalti G., Giguere P.M., Katritch V., Huang X.P., Thompson A.A., Cherezov V., Roth B.L., Stevens R.C. (2014). Molecular control of δ-opioid receptor signalling. Nature.

[37] Pepin M.C., Yue S.Y., Roberts E., Wahlestedt C., Walker P. (1997). Novel “restoration of function” mutagenesis strategy to identify amino acids of the delta-opioid receptor involved in ligand binding. J. Biol. Chem..

[38] Clayton C.C., Bruchas M.R., Lee M.L., Chavkin C. (2010). Phosphorylation of the mu-opioid receptor at tyrosine 166 (Tyr3.51) in the DRY motif reduces agonist efficacy. Mol. Pharmacol..

